# The solar eclipse: a natural meteorological experiment

**DOI:** 10.1098/rsta.2015.0225

**Published:** 2016-09-28

**Authors:** R. Giles Harrison, Edward Hanna

**Affiliations:** 1 Department of Meteorology, University of Reading, PO Box 239, Reading RG6 6BB, UK; 2 Department of Geography, University of Sheffield, Winter Street, Sheffield S10 2TN, UK

**Keywords:** eclipse meteorology, weather forecasting, science outreach, citizen science, renewable power generation

## Abstract

A solar eclipse provides a well-characterized reduction in solar radiation, of calculable amount and duration. This captivating natural astronomical phenomenon is ideally suited to science outreach activities, but the predictability of the change in solar radiation also provides unusual conditions for assessing the atmospheric response to a known stimulus. Modern automatic observing networks used for weather forecasting and atmospheric research have dense spatial coverage, so the quantitative meteorological responses to an eclipse can now be evaluated with excellent space and time resolution. Numerical models representing the atmosphere at high spatial resolution can also be used to predict eclipse-related changes and interpret the observations. Combining the models with measurements yields the elements of a controlled atmospheric experiment on a regional scale (10–1000 km), which is almost impossible to achieve by other means. This modern approach to ‘eclipse meteorology’ as identified here can ultimately improve weather prediction models and be used to plan for transient reductions in renewable electricity generation. During the 20 March 2015 eclipse, UK electrical energy demand increased by about 3 GWh (11 TJ) or about 4%, alongside reductions in the wind and photovoltaic electrical energy generation of 1.5 GWh (5.5 TJ).

This article is part of the themed issue ‘Atmospheric effects of solar eclipses stimulated by the 2015 UK eclipse’.

## 1. Introduction

**Figure 1.**
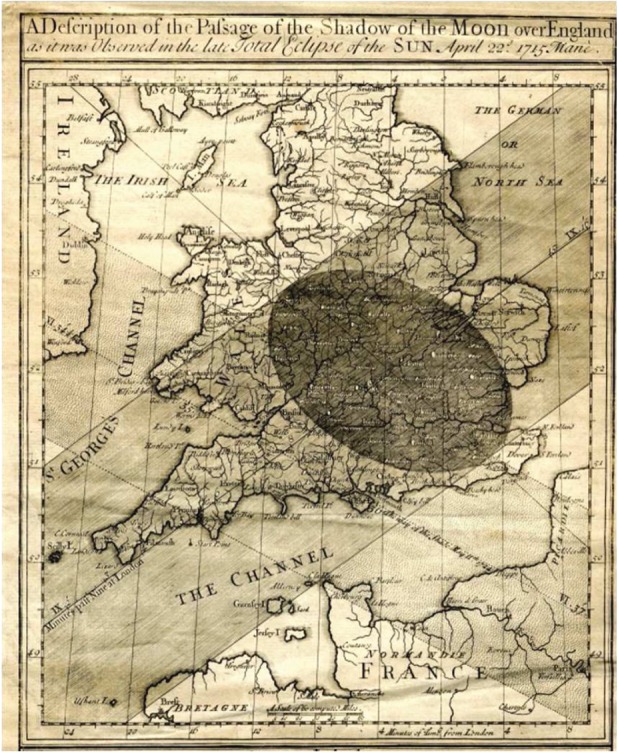
Edmund Halley’s map of the passage of the Moon’s shadow during the total eclipse of 22 April 1715 (referred to the Julian calendar). (Online version in colour.)

Eclipses have long entranced humankind as an astronomical spectacle, and yield a unique set of circumstances for disparate scientific studies. The Danish astronomer Ole Rømer used an eclipse of Jupiter’s moon Io to make the first estimate of the speed of light [1], and Sir Arthur Eddington exploited the 1919 solar eclipse for his famous test of Einstein’s theory of General Relativity [2]. Solar eclipses have provided valuable opportunities to investigate the solar atmosphere, but the studies they also permit of the terrestrial atmosphere have historically received much less attention. An atmospheric response is implicitly acknowledged in Edmund Halley’s report of the 1715 eclipse (figure 1), where he records, ‘I forbear to mention the chill and damp which attended the darkness of this eclipse of which most spectators were sensible’ [3], p. 261. However, quantitative atmospheric responses were not reported until the 1830s, with a great increase in related research following eclipses in 1980 and 1999 [4]. A modern application of eclipse meteorology in populated regions is in estimating loading of the electrical power network, as, following the expansion of wind and solar generation capability, a solar eclipse transiently reduces the generation from these renewable sources during an increase in electrical demand.

A solar eclipse’s value for atmospheric science lies in the accurate predictability of its magnitude and duration. This means that the response of the atmosphere to a well-characterized stimulus can be observed and evaluated. The more common requirement of non-eclipse experiments in atmospheric science is the need to observe effects in the presence of multiple related or unrelated changes. These complicate the interpretation, as different driving effects are often difficult to disentangle. Even so, for the solar eclipse to offer a genuine atmospheric experiment with the possibility of theoretical interpretation using numerical models, observations with dense spatial coverage and accurate, sensitive instrumentation are also likely to be required, which implies the lunar shadow will need to pass over a populated region. In part, this may explain why the potential for atmospheric and meteorological investigations has only been recognized relatively recently (e.g. [5]), in comparison with that for the astronomical endeavours. The pioneering work of H. Helm Clayton [6] offers the first example of a solar eclipse for a quantitative regional meteorological experiment, in synthesizing measurements from several sites made during the 28 May 1900 US eclipse to elucidate the associated lower atmospheric structures.

**Figure 2.**
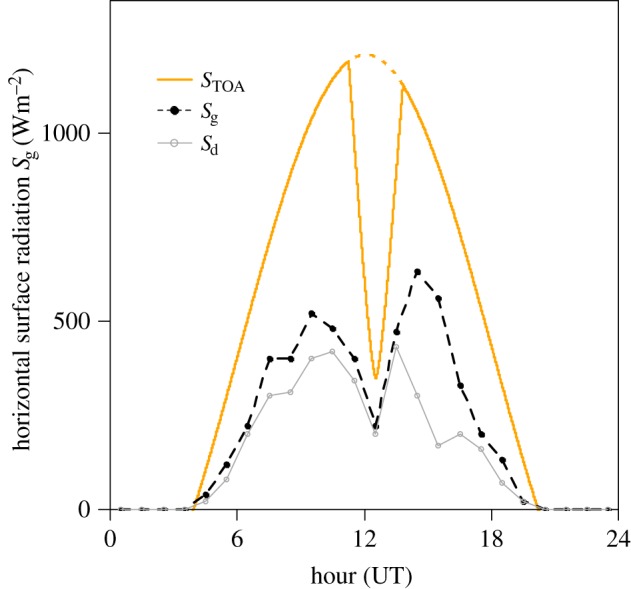
Top of atmosphere solar radiation (*S*_*TOA*_, solid line) calculated for London, UK, on 30 June 1954 (see also [7]), during the partial solar eclipse which had its maximum obscuration at London of 71% at 1233 UT. The dashed black and thin grey solid line show hourly measurements of the diffuse (*S*_d_) and total (*S*_g_) solar irradiance made by the Met Office at their Kew Observatory, near London.

A great deal more is now possible in terms of automatic monitoring of the atmospheric response to a solar eclipse, which, through its combination with numerical modelling, marks a clear new direction for eclipse meteorology from solely observational studies. The use of recording technology is not in itself new—an early example of automatic solar radiation measurements made at Kew Observatory near London, UK, during the 1954 eclipse over the southern UK is shown in figure 2, and displays the characteristic ‘bite out’ of the diurnal cycle—but the ready availability of sophisticated, often cheaper modern instrumentation and the immediate connectivity provided by the Internet yields observation networks with much greater site density and data rates [8]. Such networks exist for different purposes: they are operated by meteorological services in order to characterize the state of the atmosphere for weather forecasting models, or by local authorities for routine monitoring purposes, or sometimes otherwise by self-funded amateurs or enthusiasts (citizen scientists). The value of the latter *ad hoc* activity for studying a solar eclipse was even recognized by Edmund Halley [3, p. 261], who reports that, ‘…I have added the following synopsis of such observations as have hitherto come to my hands, acknowledging the favour of those who have been willing to promote our endeavours’.

The modern abundance of measurement networks means that vastly more atmospheric data than ever can now be obtained during solar eclipses, and there are good prospects for combining multiple sources rapidly, although effectively marshalling and combining these sources can pose its own challenges. The net result is that the surface atmospheric response to a solar eclipse can be measured at an unprecedented spatial resolution. Satellite and airborne observations of many atmospheric parameters are available, as are detailed numerical models which describe the evolution of the atmosphere with time using the laws of motion and thermodynamics, in which the effect of an eclipse can be represented or ignored. The availability of modern high spatial resolution numerical models, satellite data, *in situ* atmospheric data and extensive surface measurement networks allows detailed investigation of the atmosphere’s eclipse response. As a result, it has become timely to exploit the solar eclipse fully as a unique atmospheric experiment, in which theoretical understanding of the atmosphere can be compared with spatial measurements of the response to a brief interruption in the solar radiation. Such validation and verification leads to improvement in atmospheric models, which have great societal benefits from providing accurate weather prediction.

Continuing the long heritage of *Philosophical Transactions of the Royal Society A* in reporting the scientific use of eclipses as an experimental tool (e.g. [1–3]), this special theme issue presents a collection of investigations of the 20 March 2015 solar eclipse, in which the lunar shadow tracked through the North Atlantic to yield a substantial partial eclipse in the UK and Iceland, with totality viewable from the Faroe Islands and Svalbard. Stimulated by this event, the combination of papers presented seeks to address the broader aspects and utility of a solar eclipse, through including an essay on the depictions of solar eclipses in art [9] alongside a review of the existing understanding of eclipse meteorology [4]. In addition, Portas *et al.* [10] describe the use of the 20 March 2015 solar eclipse for science outreach activity, using a citizen science approach to generate a bespoke observing network; the related science results are analysed separately by Barnard *et al.* [11]. Multiple conventional measurement systems are employed to analyse eclipse-related changes in surface meteorological [12–16]; 17, sounding balloon [7], satellite [18] and ionospheric data [19]. Atmospheric modelling studies to underpin interpretation of the observations for advancing conceptual understanding are provided by Clark [20] and Gray & Harrison [21].

This work briefly summarizes the findings of this set of papers and further investigates the societal impact of the 20 March 2015 eclipse by evaluating its effect on electrical power generation in the UK.

## 2. Experimental eclipse studies

### (a) The influence of eclipses

Solar eclipses have long been recognized and revered by civilizations in the ancient and modern world. The depiction of solar eclipses in western art gives one example of their historical recognition as significant phenomena, which is addressed in the paper by Blatchford [9]. After their early portrayal as indicators of events of mystical importance, eclipses represented in art from the Renaissance and the Enlightenment were linked with emerging scientific knowledge of the related astronomical phenomena, such as the diamond ring effect around the Moon. Eclipses are also represented in more modern art, such as in a railway travel poster of the 1920s. The inspiration presented by an eclipse is therefore common to the artist and scientist alike, and implicitly offers a vehicle for outreach. Portas *et al.* [10] describe their use of the 20 March 2015 eclipse for a national science outreach activity—the National Eclipse Weather Experiment (NEWEx). Using a range of promotion methods building on the existing networks of learned societies, BBC Education and BBC Stargazing Live, they report that up to 3500 participants joined the project to record eclipse-induced weather changes across the UK from Cornwall to Shetland. To maintain momentum with the participants, basic synthesis of these observations acquired on the morning of 20 March 2015 was made rapidly, and a summary of the results was disseminated using national media during the afternoon. A more detailed analysis of the NEWEx data is presented by Barnard *et al.* [11], who show that, despite the simplicity of the weather measurements of air temperature, wind and cloud made by a network of untrained citizen scientists, the results were similar to the measurements obtained by professionally coordinated operational instrument networks. One informative aspect of the 20 March 2015 eclipse in terms of science outreach was that, despite layer cloud obscuring the astronomical event for many regions of the southern UK, the temperature obtained nevertheless did indicate a change, illustrating the role in science of indirect measurement and inference. The educationally detrimental aspects of one meteorological factor were therefore partially offset by the utility of investigating another.

### (b) Surface meteorological changes

Measurement of meteorological responses to eclipses has historically been dominated by observations of surface changes, principally in temperature [4]. However, there is also interest in the surface wind speed and direction and surface pressure, because these quantities provide essential information about the lower atmosphere’s structure [22].

As remarked earlier, the spatial resolution of modern surface measurement networks having rapid sampling rates is unprecedented, which is reflected in the studies presented. For comparison, in their analysis of the UK eclipse of 11 August 1999, both Hanna [23] and Gray & Harrison [24] used hourly resolution data from up to 121 UK meteorological stations. For the 20 March 2015 eclipse, Hanna *et al.* [12] used 1 min data from 76 UK sites, with an additional 30 in the Faroes and 148 in Iceland. Clark [13] employed 1 min data from the UK’s Meteorological Monitoring System (MMS), amounting to 266 measuring sites. Gray & Harrison [21] included analysis of measurements from a further independent network of 868 roadside sites, providing air temperature, wind speed and direction at hourly resolution or better. The NEWEx citizen science system generated some 15 000 observations of air temperature, cloudiness, and wind speed and direction from 309 locations across the UK, during a 3 h window centred on the time of peak eclipse [11].

Broadly consistent patterns existed in the observations across the UK from these different measurement systems, although changes in the synoptic conditions during the eclipse complicated the interpretation in some regions. Hanna *et al.* [12] showed a mean reduction in temperature ranging from 0.31°C in cloudy conditions to 0.91°C in clear conditions, with the minimum lagging the solar radiation minimum by 10 min. Clark [13] found a median temperature reduction of 1.02°C, which lagged the solar radiation minimum by 15 min. Further, Gray & Harrison [21] found consistency between roadside wind and near-surface temperature data and that from the Met Office sites. Hanna *et al.* [12] reported a 9% mean reduction in wind speed, and found no evidence of surface pressure change, and Clark [13] likewise reported a statistically significant slackening of the wind. The difference in the eclipse-induced temperature drop between cloudy and clear conditions was between 0.6°C [12] and 1.6°C [13] using different methodologies. For inland regions without cloud where eclipse-induced effects were greatest, Gray & Harrison [21] reported reductions in wind speed together with an anticlockwise change in wind direction.

An alternative source of surface data covering a region is to use satellite remote sensing. Good [18] used data from the SEVIRI satellite to show the variation in land surface temperature. The greatest reduction in temperatures occurred in the cloud-free region of the central UK, which is consistent with the spatial pattern in the near-surface air temperatures reported by Hanna *et al.* [12] and Clark [13].

Many other parameters have previously been studied at a single site during an eclipse (e.g. [4,22]). Burt [14] and Bennett [15] analysed the wide range of measurements made at the Reading University Atmospheric Observatory, UK, where the eclipse reduced the solar radiation by 85% but the measurements were obtained under a 400 m thick layer cloud with its base at about 200 m above the surface. During the 2015 eclipse, Burt [14] found a reduction in near-surface turbulence and a reduction in cloud base height. This is partly consistent with results from the 11 August 1999 eclipse over southeast England where decreased convection was observed and convective cloud dissipated [23]. However, Bennett [15] concluded that the reduction in turbulence was insufficient to influence the surface atmospheric electric field.

### (c) Upper air measurements

Much as an eclipse yields predictable and rapid changes in the solar radiation environment, the presence of cloud can prevent accurate measurements of ‘ideal’ (theoretically calculated) eclipse-induced solar radiation changes, although there is obviously still significant darkening. One approach is to position the solar radiation detectors above the cloud. Modified weather balloons offer an inexpensive platform to carry photodiode sensors to provide such data during a typical meteorological balloon flight of approximately 2 h duration. Harrison *et al.* [7] describe a coordinated campaign of balloon-carried solar radiation sensors from launch sites straddling the path of the 20 March 2015 eclipse, at Reading, UK (51.44° N, 0.94° W), Lerwick, UK (60.15° N, 1.13° W), and Reykjavik, Iceland (64.13° N, 21.90° W). All three balloons reached sufficient altitude above the cloud to demonstrate agreement of the measured eclipse-induced solar radiation changes with theory. Marlton *et al.* [16] used the soundings from Reading and Lerwick to search for eclipse-induced gravity waves in the troposphere, which have previously been associated with some eclipses [4]. Despite observations of pressure fluctuations, the complexity of the synoptic conditions at the time and the island topography at Lerwick precluded the fluctuations being unambiguously attributed to the eclipse.

### (d) Ionospheric measurements

Detectable changes occur in the upper atmosphere from a solar eclipse. Scott *et al.* [19] showed eclipse-induced changes in the cut-off frequency of the ionosphere’s E layer (at 100 km) above Chilton, UK, during 20 March 2015, and compared them with simultaneous changes in solar ultraviolet and X-ray emissions. This provides a calibration for the long series of ionosonde measurements which began in the UK in 1932, which enables solar disc properties to be inferred from eclipse-influenced historical ionosonde data.

### (e) Modelling

Numerical models of the atmospheric circulation provide detailed three-dimensional information concerning the evolution of wind fields and temperatures. Such models can be configured to include the influence of an eclipse, or used to predict the changes which would have occurred in the absence of an eclipse. Differencing results from the same model with and without knowledge of the eclipse included allows predictions of the eclipse-induced changes to be made, or the observations made can be differenced from the predictions of an eclipse-ignorant model to determine the eclipse-induced effects. The second approach was used by Gray & Harrison [24] for studying the 1999 eclipse, and this was undertaken again for the 20 March 2015 eclipse in Gray & Harrison [21], but with a much more extensive set of measurements for comparison. The first approach was used by Clark [20] with a nested arrangement of Met Office models to provide predictions at 1.5 km horizontal resolution over the UK. Both modelling studies show small anticlockwise changes in the wind direction in some areas, which are associated with near-surface changes in atmospheric mixing.

## 3. Electrical power generation considerations

Solar eclipses briefly influence the production of electricity from photovoltaic (PV) and wind generation. The proportion of electrical energy provided by renewable sources such as that from large arrays of PV cells and wind turbines has increased considerably in recent decades, particularly in wind generation. In 2013 about 21% of global energy generation was produced from renewable sources, which is estimated to reach 25% in 2040 [25]. Some countries have made determined obligations to achieve an increase in the fraction of electricity generated from renewables, for example: Ireland is committed to produce at least 40% of all electrical energy consumed from renewable sources by 2020 [26]. While solar eclipses are relatively rare events—Earth experienced 228 eclipses during the twentieth century [27]—the global increase in the contribution of solar and wind sources to electricity generation means that the transient effect of solar eclipses on electricity generation will need to be considered more often.

The demand for electricity varies during the day, from day to day and across the week, for which predictions of the likely load are made to plan the generation needed to maintain a stable supply network. The timing of a solar eclipse is of course known in advance, but the actual effects on generation and demand will be weather dependent. The studies of eclipse meteorology provide information to apply to this problem, together with experience obtained from previous eclipse effects on power generation. For example, in the 1999 solar eclipse, when there was a smaller contribution from renewable sources, the UK electrical power demand was reduced as people stopped what they were doing and went to watch the eclipse in mostly clear skies (J. Caplin 2016, personal communication).

**Figure 3.**
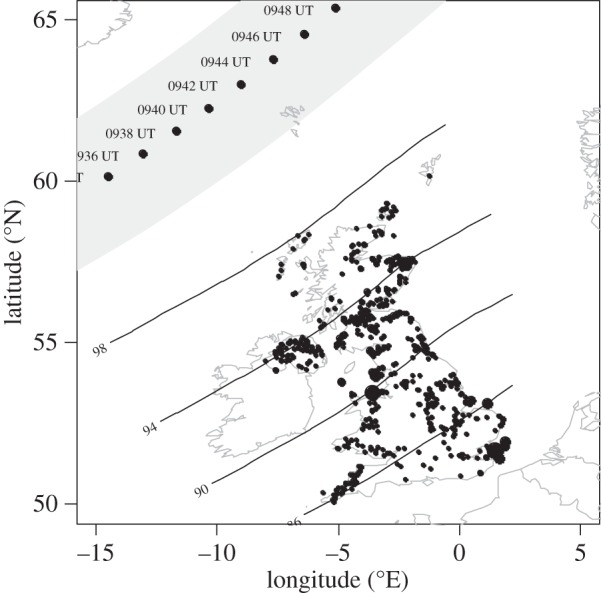
Distribution of UK electrical generation sites operating by wind power, with their positions marked by black points having radii proportional to their generating capacity (from [28]). The region of totality of the solar eclipse of 20 March 2015 is shown as a grey band with the times of totality marked, and contours of percentage obscuration are drawn for the regions experiencing a partial eclipse.

For the 20 March 2015 solar eclipse, more of the UK was overcast than for the 1999 event and the proportion of renewable electrical generation increased. Figure 3 shows the position of the major wind turbine sites in the UK, together with the regional effect of the 20 March 2015 eclipse. From this distribution of wind turbine sites, it is clear that changes in the wind due to the eclipse had a significant potential to influence generation. (The distribution of the solar generation sites is not shown, but these contribute less power in total than wind generation, and are mainly in the south of the UK, which experienced less solar obscuration than the north.)

**Figure 4.**
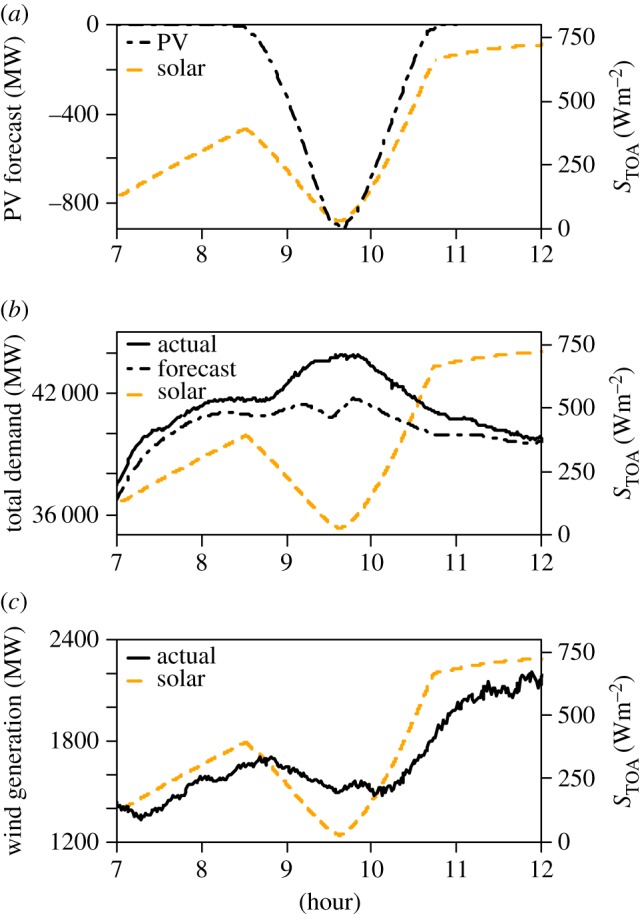
National grid electricity variations associated with the 20 March 2015 solar eclipse. Time series (in hours UT) of (*a*) forecast expected drop in photovoltaic (PV) generation, (*b*) forecast and measured total demand of electricity and (*c*) measured variation in wind generation. (All panels also show the calculated change in solar radiation received by a horizontal surface at the top of atmosphere, *S*_*TOA*_, following [7] as the orange dashed line; measured power grid data in (*b*,*c*) were sampled at 1 min intervals.)

Figure 4 shows measurements and predictions of energy demand in the UK National Grid during the 20 March 2015 solar eclipse. Figure 4*a* shows the prediction of the reduction in PV generation, on the basis of a direct and unlagged response to the reduction in incoming solar radiation. The effect of the PV reduction is to draw more power from the National Grid, together with other eclipse-related additional demand, such as for lighting and heating. Figure 4*b* shows the increase in total demand, compared with a forecast based on demand from the previous 2 days. Some of this increase in demand also comes from a reduction in wind generation. Figure 4*c* shows the reduction in wind generation recorded during the solar eclipse. This response is lagged on the minimum in the solar response, by approximately 30 minutes. Clark [13] remarked on the effects of coastal topography on the time response in the wind flow, which may have been a factor at some of the coastal generating sites.

The integrated effect of the solar eclipse on UK electrical energy generation has been evaluated by assuming an otherwise linear increase in generation between the beginning and end of the eclipse (between 0848 UT and 1100 UT on 20 March 2015), and is summarized in table 1. This shows that the change in wind contribution is important to allow for, as the reduction in energy from less wind generation amounts to half as much again in terms of the reduction in energy from the loss of PV generation. Although the combination of wind and PV reductions constitutes only about 4% of the concurrent UK national demand for an eclipse, which is a rare event, the identifiable response is useful in electrical supply system planning for other meteorological fluctuations.

**Table 1.** Forecast and observed changes in UK electrical demand and generations from 0848 UT to 1100 UT during the 20 March 2015 partial solar eclipse.

	energy (MWh)
forecast change in PV generation	−1020
measured change in wind generation	−510
measured change in demand on the National Grid	3040
total demand on the National Grid during eclipse	95 116

## 4. Conclusion

As an astronomical phenomenon an eclipse is almost perfectly predictable from orbital parameters far in advance of the event itself. In contrast, the meteorological conditions and response, which determine the viewing conditions of the eclipse, are far less predictable even until a short time beforehand. The detail of the meteorological response itself, obtained through observations made over a wide area, provides direct information with which to test weather prediction models, improvements in which have much greater societal benefits than just the prediction of eclipse viewing conditions. Such results from a natural experiment represent an important impact of the study of eclipse meteorology. In addition, as a scientific outreach opportunity, an eclipse offers excellent prospects for motivating interested individuals in the viewing zone. For the 20 March 2015 eclipse, which was effectively the first major UK solar eclipse of the social media era, successful and enthusiastic nationwide engagement was obtained.

Comparison of models and measurements during the 20 March 2015 eclipse indicate that, beyond the relatively well-predicted changes in temperature in regions with and without cloud, there are consistent changes in wind speed and direction which may provide a partial explanation for the various and disparate suggestions of eclipse-related changes in wind. Although the conceptual picture of Clayton [6] has provided the primary explanatory framework for this possibility, the limitations of this ‘eclipse cyclone’ interpretation, based entirely on surface measurements, are becoming clear. High-resolution numerical simulations show instead that the wind effects can be related to eclipse-induced changes in the atmospheric boundary layer where the surface interacts strongly with the mean flow, and the wind direction is affected by drag over the surface.

Beyond the verification and improvement of weather prediction models from the advances in basic understanding of atmospheric phenomena, eclipse meteorology provides a predictive capability for electric power generation networks. The effect of reduction in solar generation is the most obvious quantity to predict, but the effect of wind speed reductions also has to be anticipated. In the UK for the 20 March 2015 eclipse, the reduction in wind generation was half as much again as that from the reduction in PV generation. The combination of eclipse-aware atmospheric models with well-characterized knowledge of generating networks offers promise in testing predictions of changes in national electrical generation from meteorological fluctuations. Because of the expansion in the use of renewables, the need to correctly balance naturally induced transient changes in electricity demand and generation will only increase. In advancing this and the refinement of weather prediction models of great importance to society, the opportunities of the solar eclipse as a unique natural experiment should not be neglected.

## Supplementary Material

ElectricityData_Electronic Supplementary Material

## Data accessibility

The data for figure 2 exist in the Met Office Integrated Data Archive System (MIDAS) Land and Marine Surface Stations Data (1853-current), provided by the NCAS British Atmospheric Data Centre. (http://catalogue.ceda.ac.uk/uuid/220a65615218d5c9cc9e4785a3234bd0). The National Grid data values on which figure 4 is based are available in the electronic supplementary material.
