# Supplementary material for: The solar eclipse: a natural meteorological experiment
Source: Philos Trans A Math Phys Eng Sci. 2016 Sep 28;374(2077):20150225. doi: 10.1098/rsta.2015.0225 (PMC5004055; doi:10.1098/rsta.2015.0225)
Supplement: ElectricityData_Electronic Supplementary Material [file rsta20150225supp1.doc]

# National Grid data - 20^th^ March 2015 eclipse

UK electrical demand, wind power generation and photovoltaic (PV) power generation, as forecast and metered during the 20^th^ March 2015 solar eclipse. These data values from the National Grid were used to produce figure 4 in the paper.

| date and time | PV change | Demand | Demand | Wind |
| --- | --- | --- | --- | --- |
|  | forecast | forecast | metered | metered |
|  | (MW) | (MW) | (MW) | (MW) |
| 20/03/2015 07:00:00 | 0 | 36789 | 37553 | 1417 |
| 20/03/2015 07:01:00 | 0 | 36919 | 37618 | 1412 |
| 20/03/2015 07:02:00 | 0 | 37049 | 37719 | 1422 |
| 20/03/2015 07:03:00 | 0 | 37179 | 37877 | 1393 |
| 20/03/2015 07:04:00 | 0 | 37309 | 38046 | 1406 |
| 20/03/2015 07:05:00 | 0 | 37439 | 38172 | 1385 |
| 20/03/2015 07:06:00 | 0 | 37559 | 38379 | 1410 |
| 20/03/2015 07:07:00 | 0 | 37679 | 38542 | 1405 |
| 20/03/2015 07:08:00 | 0 | 37800 | 38720 | 1383 |
| 20/03/2015 07:09:00 | 0 | 37920 | 38854 | 1393 |
| 20/03/2015 07:10:00 | 0 | 38040 | 38990 | 1390 |
| 20/03/2015 07:11:00 | 0 | 38143 | 39172 | 1381 |
| 20/03/2015 07:12:00 | 0 | 38246 | 39222 | 1355 |
| 20/03/2015 07:13:00 | 0 | 38350 | 39350 | 1368 |
| 20/03/2015 07:14:00 | 0 | 38453 | 39481 | 1350 |
| 20/03/2015 07:15:00 | 0 | 38556 | 39501 | 1338 |
| 20/03/2015 07:16:00 | 0 | 38637 | 39608 | 1356 |
| 20/03/2015 07:17:00 | 0 | 38718 | 39605 | 1336 |
| 20/03/2015 07:18:00 | 0 | 38800 | 39790 | 1346 |
| 20/03/2015 07:19:00 | 0 | 38881 | 39851 | 1355 |
| 20/03/2015 07:20:00 | 0 | 38962 | 39854 | 1368 |
| 20/03/2015 07:21:00 | 0 | 39032 | 39853 | 1360 |
| 20/03/2015 07:22:00 | 0 | 39101 | 39942 | 1357 |
| 20/03/2015 07:23:00 | 0 | 39171 | 40029 | 1361 |
| 20/03/2015 07:24:00 | 0 | 39240 | 40115 | 1378 |
| 20/03/2015 07:25:00 | 0 | 39310 | 40142 | 1387 |
| 20/03/2015 07:26:00 | 0 | 39370 | 40144 | 1387 |
| 20/03/2015 07:27:00 | 0 | 39429 | 40189 | 1396 |
| 20/03/2015 07:28:00 | 0 | 39489 | 40167 | 1381 |
| 20/03/2015 07:29:00 | 0 | 39548 | 40240 | 1394 |
| 20/03/2015 07:30:00 | 0 | 39608 | 40228 | 1397 |
| 20/03/2015 07:31:00 | 0 | 39660 | 40185 | 1392 |
| 20/03/2015 07:32:00 | 0 | 39711 | 40204 | 1412 |
| 20/03/2015 07:33:00 | 0 | 39763 | 40279 | 1416 |
| 20/03/2015 07:34:00 | 0 | 39814 | 40290 | 1419 |
| 20/03/2015 07:35:00 | 0 | 39866 | 40328 | 1430 |
| 20/03/2015 07:36:00 | 0 | 39912 | 40345 | 1448 |
| 20/03/2015 07:37:00 | 0 | 39958 | 40439 | 1443 |
| 20/03/2015 07:38:00 | 0 | 40004 | 40509 | 1455 |
| 20/03/2015 07:39:00 | 0 | 40050 | 40569 | 1473 |
| 20/03/2015 07:40:00 | 0 | 40096 | 40638 | 1468 |
| 20/03/2015 07:41:00 | 0 | 40138 | 40677 | 1470 |
| 20/03/2015 07:42:00 | 0 | 40180 | 40726 | 1467 |
| 20/03/2015 07:43:00 | 0 | 40223 | 40817 | 1478 |
| 20/03/2015 07:44:00 | 0 | 40265 | 40791 | 1452 |
| 20/03/2015 07:45:00 | 0 | 40307 | 40871 | 1476 |
| 20/03/2015 07:46:00 | 0 | 40346 | 38678 | 1473 |
| 20/03/2015 07:47:00 | 0 | 40385 | 40874 | 1478 |
| 20/03/2015 07:48:00 | 0 | 40423 | 40969 | 1496 |
| 20/03/2015 07:49:00 | 0 | 40462 | 40985 | 1511 |
| 20/03/2015 07:50:00 | 0 | 40501 | 41100 | 1527 |
| 20/03/2015 07:51:00 | 0 | 40534 | 41079 | 1543 |
| 20/03/2015 07:52:00 | 0 | 40567 | 41145 | 1545 |
| 20/03/2015 07:53:00 | 0 | 40599 | 41181 | 1541 |
| 20/03/2015 07:54:00 | 0 | 40632 | 41261 | 1554 |
| 20/03/2015 07:55:00 | 0 | 40665 | 41218 | 1559 |
| 20/03/2015 07:56:00 | 0 | 40692 | 41307 | 1551 |
| 20/03/2015 07:57:00 | 0 | 40719 | 41238 | 1565 |
| 20/03/2015 07:58:00 | 0 | 40745 | 41246 | 1571 |
| 20/03/2015 07:59:00 | 0 | 40772 | 41344 | 1588 |
| 20/03/2015 08:00:00 | 0 | 40799 | 41344 | 1574 |
| 20/03/2015 08:01:00 | 0 | 40820 | 41418 | 1575 |
| 20/03/2015 08:02:00 | 0 | 40842 | 41514 | 1587 |
| 20/03/2015 08:03:00 | 0 | 40863 | 41502 | 1569 |
| 20/03/2015 08:04:00 | 0 | 40885 | 41512 | 1584 |
| 20/03/2015 08:05:00 | 0 | 40906 | 41631 | 1578 |
| 20/03/2015 08:06:00 | 0 | 40922 | 41614 | 1573 |
| 20/03/2015 08:07:00 | 0 | 40938 | 41620 | 1579 |
| 20/03/2015 08:08:00 | 0 | 40955 | 41580 | 1560 |
| 20/03/2015 08:09:00 | 0 | 40971 | 41658 | 1557 |
| 20/03/2015 08:10:00 | 0 | 40987 | 41568 | 1562 |
| 20/03/2015 08:11:00 | 0 | 40998 | 41624 | 1574 |
| 20/03/2015 08:12:00 | 0 | 41009 | 41666 | 1572 |
| 20/03/2015 08:13:00 | 0 | 41021 | 41668 | 1581 |
| 20/03/2015 08:14:00 | 0 | 41032 | 41681 | 1570 |
| 20/03/2015 08:15:00 | 0 | 41043 | 41601 | 1582 |
| 20/03/2015 08:16:00 | 0 | 41044 | 41663 | 1573 |
| 20/03/2015 08:17:00 | 0 | 41045 | 41706 | 1573 |
| 20/03/2015 08:18:00 | 0 | 41045 | 41672 | 1586 |
| 20/03/2015 08:19:00 | 0 | 41046 | 41727 | 1598 |
| 20/03/2015 08:20:00 | 0 | 41047 | 41714 | 1597 |
| 20/03/2015 08:21:00 | 1 | 41038 | 41724 | 1595 |
| 20/03/2015 08:22:00 | 1 | 41030 | 41695 | 1606 |
| 20/03/2015 08:23:00 | 2 | 41021 | 41626 | 1620 |
| 20/03/2015 08:24:00 | 3 | 41013 | 41715 | 1629 |
| 20/03/2015 08:25:00 | 4 | 41004 | 41731 | 1646 |
| 20/03/2015 08:26:00 | 4 | 40990 | 41729 | 1657 |
| 20/03/2015 08:27:00 | 5 | 40976 | 41674 | 1640 |
| 20/03/2015 08:28:00 | 6 | 40963 | 41737 | 1660 |
| 20/03/2015 08:29:00 | 7 | 40949 | 41662 | 1661 |
| 20/03/2015 08:30:00 | 7 | 40935 | 41625 | 1656 |
| 20/03/2015 08:31:00 | 13 | 40920 | 41639 | 1650 |
| 20/03/2015 08:32:00 | 19 | 40905 | 41656 | 1653 |
| 20/03/2015 08:33:00 | 26 | 40891 | 41671 | 1662 |
| 20/03/2015 08:34:00 | 32 | 40876 | 41649 | 1654 |
| 20/03/2015 08:35:00 | 38 | 40861 | 41706 | 1662 |
| 20/03/2015 08:36:00 | 44 | 40849 | 41723 | 1646 |
| 20/03/2015 08:37:00 | 50 | 40838 | 41732 | 1661 |
| 20/03/2015 08:38:00 | 56 | 40826 | 41741 | 1667 |
| 20/03/2015 08:39:00 | 62 | 40815 | 41618 | 1676 |
| 20/03/2015 08:40:00 | 68 | 40803 | 41634 | 1686 |
| 20/03/2015 08:41:00 | 79 | 40799 | 41672 | 1699 |
| 20/03/2015 08:42:00 | 90 | 40795 | 41586 | 1672 |
| 20/03/2015 08:43:00 | 101 | 40791 | 41718 | 1679 |
| 20/03/2015 08:44:00 | 112 | 40787 | 41622 | 1689 |
| 20/03/2015 08:45:00 | 123 | 40783 | 41704 | 1660 |
| 20/03/2015 08:46:00 | 134 | 40792 | 41762 | 1692 |
| 20/03/2015 08:47:00 | 144 | 40801 | 41660 | 1681 |
| 20/03/2015 08:48:00 | 155 | 40810 | 41717 | 1693 |
| 20/03/2015 08:49:00 | 166 | 40819 | 41734 | 1704 |
| 20/03/2015 08:50:00 | 177 | 40828 | 41775 | 1691 |
| 20/03/2015 08:51:00 | 192 | 40843 | 41858 | 1691 |
| 20/03/2015 08:52:00 | 206 | 40859 | 41981 | 1674 |
| 20/03/2015 08:53:00 | 220 | 40874 | 41988 | 1664 |
| 20/03/2015 08:54:00 | 235 | 40890 | 42049 | 1660 |
| 20/03/2015 08:55:00 | 249 | 40905 | 42062 | 1651 |
| 20/03/2015 08:56:00 | 263 | 40924 | 42141 | 1652 |
| 20/03/2015 08:57:00 | 278 | 40942 | 42182 | 1641 |
| 20/03/2015 08:58:00 | 292 | 40961 | 42343 | 1638 |
| 20/03/2015 08:59:00 | 306 | 40979 | 42388 | 1645 |
| 20/03/2015 09:00:00 | 321 | 40998 | 42506 | 1641 |
| 20/03/2015 09:01:00 | 338 | 41017 | 42542 | 1642 |
| 20/03/2015 09:02:00 | 356 | 41035 | 42593 | 1622 |
| 20/03/2015 09:03:00 | 373 | 41054 | 42619 | 1613 |
| 20/03/2015 09:04:00 | 390 | 41072 | 42798 | 1631 |
| 20/03/2015 09:05:00 | 408 | 41091 | 42821 | 1637 |
| 20/03/2015 09:06:00 | 425 | 41106 | 43011 | 1618 |
| 20/03/2015 09:07:00 | 443 | 41122 | 42905 | 1630 |
| 20/03/2015 09:08:00 | 460 | 41137 | 42860 | 1609 |
| 20/03/2015 09:09:00 | 478 | 41153 | 42998 | 1605 |
| 20/03/2015 09:10:00 | 495 | 41168 | 43024 | 1600 |
| 20/03/2015 09:11:00 | 516 | 41177 | 43145 | 1590 |
| 20/03/2015 09:12:00 | 537 | 41186 | 43250 | 1604 |
| 20/03/2015 09:13:00 | 558 | 41196 | 43188 | 1593 |
| 20/03/2015 09:14:00 | 579 | 41205 | 43263 | 1585 |
| 20/03/2015 09:15:00 | 600 | 41214 | 43351 | 1574 |
| 20/03/2015 09:16:00 | 620 | 41215 | 43438 | 1572 |
| 20/03/2015 09:17:00 | 641 | 41215 | 43498 | 1561 |
| 20/03/2015 09:18:00 | 662 | 41216 | 43479 | 1559 |
| 20/03/2015 09:19:00 | 683 | 41216 | 43540 | 1561 |
| 20/03/2015 09:20:00 | 704 | 41217 | 43589 | 1571 |
| 20/03/2015 09:21:00 | 721 | 41214 | 43601 | 1567 |
| 20/03/2015 09:22:00 | 738 | 41210 | 43607 | 1556 |
| 20/03/2015 09:23:00 | 755 | 41207 | 43578 | 1549 |
| 20/03/2015 09:24:00 | 772 | 41203 | 43619 | 1562 |
| 20/03/2015 09:25:00 | 788 | 41200 | 43614 | 1546 |
| 20/03/2015 09:26:00 | 805 | 41194 | 43603 | 1546 |
| 20/03/2015 09:27:00 | 822 | 41187 | 43618 | 1535 |
| 20/03/2015 09:28:00 | 839 | 41181 | 43616 | 1528 |
| 20/03/2015 09:29:00 | 856 | 41174 | 43593 | 1518 |
| 20/03/2015 09:30:00 | 873 | 41168 | 43615 | 1531 |
| 20/03/2015 09:31:00 | 877 | 41159 | 43702 | 1520 |
| 20/03/2015 09:32:00 | 881 | 41150 | 43734 | 1518 |
| 20/03/2015 09:33:00 | 886 | 41141 | 43759 | 1505 |
| 20/03/2015 09:34:00 | 890 | 41132 | 43756 | 1514 |
| 20/03/2015 09:35:00 | 894 | 41123 | 43871 | 1496 |
| 20/03/2015 09:36:00 | 898 | 41113 | 43750 | 1491 |
| 20/03/2015 09:37:00 | 902 | 41103 | 43794 | 1498 |
| 20/03/2015 09:38:00 | 907 | 41092 | 43886 | 1506 |
| 20/03/2015 09:39:00 | 911 | 41082 | 43878 | 1499 |
| 20/03/2015 09:40:00 | 915 | 41072 | 43781 | 1512 |
| 20/03/2015 09:41:00 | 907 | 41061 | 43913 | 1530 |
| 20/03/2015 09:42:00 | 900 | 41050 | 43860 | 1510 |
| 20/03/2015 09:43:00 | 892 | 41038 | 43701 | 1518 |
| 20/03/2015 09:44:00 | 885 | 41027 | 43754 | 1524 |
| 20/03/2015 09:45:00 | 877 | 41016 | 43805 | 1538 |
| 20/03/2015 09:46:00 | 870 | 41004 | 43782 | 1537 |
| 20/03/2015 09:47:00 | 862 | 40992 | 43867 | 1552 |
| 20/03/2015 09:48:00 | 855 | 40981 | 43734 | 1561 |
| 20/03/2015 09:49:00 | 847 | 40969 | 43839 | 1533 |
| 20/03/2015 09:50:00 | 840 | 40957 | 43836 | 1536 |
| 20/03/2015 09:51:00 | 824 | 40943 | 43909 | 1522 |
| 20/03/2015 09:52:00 | 809 | 40929 | 43796 | 1528 |
| 20/03/2015 09:53:00 | 794 | 40916 | 43760 | 1562 |
| 20/03/2015 09:54:00 | 778 | 40902 | 43724 | 1528 |
| 20/03/2015 09:55:00 | 763 | 40888 | 43665 | 1548 |
| 20/03/2015 09:56:00 | 748 | 40873 | 43630 | 1538 |
| 20/03/2015 09:57:00 | 732 | 40857 | 43507 | 1525 |
| 20/03/2015 09:58:00 | 717 | 40842 | 43467 | 1542 |
| 20/03/2015 09:59:00 | 702 | 40826 | 43408 | 1512 |
| 20/03/2015 10:00:00 | 686 | 40811 | 43365 | 1504 |
| 20/03/2015 10:01:00 | 669 | 40795 | 43320 | 1517 |
| 20/03/2015 10:02:00 | 651 | 40778 | 43242 | 1479 |
| 20/03/2015 10:03:00 | 633 | 40762 | 43101 | 1484 |
| 20/03/2015 10:04:00 | 616 | 40745 | 43080 | 1497 |
| 20/03/2015 10:05:00 | 598 | 40729 | 43054 | 1519 |
| 20/03/2015 10:06:00 | 580 | 40712 | 42997 | 1525 |
| 20/03/2015 10:07:00 | 563 | 40694 | 43031 | 1518 |
| 20/03/2015 10:08:00 | 545 | 40677 | 42966 | 1477 |
| 20/03/2015 10:09:00 | 527 | 40659 | 42942 | 1519 |
| 20/03/2015 10:10:00 | 510 | 40642 | 42848 | 1493 |
| 20/03/2015 10:11:00 | 492 | 40624 | 42727 | 1518 |
| 20/03/2015 10:12:00 | 474 | 40607 | 42702 | 1528 |
| 20/03/2015 10:13:00 | 457 | 40589 | 42622 | 1528 |
| 20/03/2015 10:14:00 | 439 | 40572 | 42623 | 1507 |
| 20/03/2015 10:15:00 | 421 | 40554 | 42215 | 1516 |
| 20/03/2015 10:16:00 | 404 | 40533 | 42455 | 1517 |
| 20/03/2015 10:17:00 | 386 | 40513 | 42428 | 1551 |
| 20/03/2015 10:18:00 | 369 | 40492 | 42448 | 1547 |
| 20/03/2015 10:19:00 | 351 | 40472 | 42348 | 1562 |
| 20/03/2015 10:20:00 | 333 | 40451 | 42346 | 1544 |
| 20/03/2015 10:21:00 | 316 | 40428 | 42283 | 1583 |
| 20/03/2015 10:22:00 | 299 | 40404 | 42279 | 1556 |
| 20/03/2015 10:23:00 | 281 | 40381 | 42190 | 1612 |
| 20/03/2015 10:24:00 | 264 | 40357 | 42156 | 1597 |
| 20/03/2015 10:25:00 | 246 | 40334 | 42021 | 1623 |
| 20/03/2015 10:26:00 | 229 | 40310 | 41909 | 1614 |
| 20/03/2015 10:27:00 | 212 | 40286 | 41907 | 1606 |
| 20/03/2015 10:28:00 | 194 | 40262 | 41844 | 1648 |
| 20/03/2015 10:29:00 | 177 | 40238 | 41797 | 1661 |
| 20/03/2015 10:30:00 | 160 | 40214 | 41822 | 1681 |
| 20/03/2015 10:31:00 | 146 | 40191 | 41800 | 1668 |
| 20/03/2015 10:32:00 | 133 | 40168 | 41658 | 1686 |
| 20/03/2015 10:33:00 | 120 | 40145 | 41589 | 1682 |
| 20/03/2015 10:34:00 | 107 | 40122 | 41573 | 1715 |
| 20/03/2015 10:35:00 | 93 | 40099 | 41548 | 1705 |
| 20/03/2015 10:36:00 | 80 | 40079 | 41527 | 1711 |
| 20/03/2015 10:37:00 | 67 | 40059 | 41396 | 1713 |
| 20/03/2015 10:38:00 | 53 | 40040 | 41309 | 1741 |
| 20/03/2015 10:39:00 | 40 | 40020 | 41276 | 1744 |
| 20/03/2015 10:40:00 | 27 | 40000 | 41251 | 1761 |
| 20/03/2015 10:41:00 | 24 | 39985 | 41237 | 1793 |
| 20/03/2015 10:42:00 | 22 | 39971 | 41200 | 1803 |
| 20/03/2015 10:43:00 | 19 | 39956 | 41101 | 1799 |
| 20/03/2015 10:44:00 | 16 | 39942 | 41139 | 1817 |
| 20/03/2015 10:45:00 | 13 | 39927 | 41045 | 1820 |
| 20/03/2015 10:46:00 | 11 | 39921 | 41044 | 1859 |
| 20/03/2015 10:47:00 | 8 | 39915 | 41055 | 1895 |
| 20/03/2015 10:48:00 | 5 | 39910 | 40951 | 1872 |
| 20/03/2015 10:49:00 | 3 | 39904 | 40936 | 1880 |
| 20/03/2015 10:50:00 | 0 | 39898 | 40978 | 1862 |
| 20/03/2015 10:51:00 | 0 | 39898 | 40961 | 1923 |
| 20/03/2015 10:52:00 | 0 | 39897 | 40959 | 1904 |
| 20/03/2015 10:53:00 | 0 | 39897 | 40875 | 1904 |
| 20/03/2015 10:54:00 | 0 | 39896 | 40801 | 1963 |
| 20/03/2015 10:55:00 | 0 | 39896 | 40753 | 1954 |
| 20/03/2015 10:56:00 | 0 | 39899 | 40707 | 1964 |
| 20/03/2015 10:57:00 | 0 | 39902 | 40702 | 1971 |
| 20/03/2015 10:58:00 | 0 | 39905 | 40714 | 1977 |
| 20/03/2015 10:59:00 | 0 | 39908 | 40739 | 1976 |
| 20/03/2015 11:00:00 | 0 | 39911 | 40763 | 1986 |
